# Genetic and immune profiling for potential therapeutic targets in adult human craniopharyngioma

**DOI:** 10.31487/j.COR.2019.03.05

**Published:** 2019-06-27

**Authors:** Cynthia Kassab, Daniel Zamler, Carlos Kamiya-Matsuoka, Zoran Gatalica, Joanne Xiu, David Spetzler, Amy B. Heimberger

**Affiliations:** 1 Departments of Neurosurgery, The University of Texas MD Anderson Cancer Center, Houston, TX; 2 Genomic Medicine, The University of Texas MD Anderson Cancer Center, Houston, TX; 3 Cancer Biology, The University of Texas MD Anderson Cancer Center, Houston, TX; 4 Neuro-Oncology, The University of Texas MD Anderson Cancer Center, Houston, TX; 5 Caris Life Sciences, Phoenix, AZ

**Keywords:** Craniopharyngioma, genetic profiling, immune profiling, PD-L1, EGFR

## Abstract

Craniopharyngioma is a rare tumor in adults. Although histologically benign, it can be locally aggressive and may require additional therapeutic modalities to surgical resection. Analyses including next generation sequencing, chromogenic and *in situ* hybridization, immunohistochemistry, and gene amplification were used to profile craniopharyngiomas (n=6) for frequently altered therapeutic targets. Four of six patients had the *BRAF*^*V600E*^ missense mutation, frequent in the papillary craniopharyngioma subtype. One patient had a missense mutation in the *WNT* pathway, specifically *CTNNB1*, often associated with the adamantinomatous subtype. Craniopharyngiomas lacked microsatellite instability, had low tumor mutational burden, but did express PD-L1 protein, indicating potential therapeutic value for immune checkpoint inhibition. We identified mutations not previously described, including an *E318K* missense mutation in the *MITF* gene, an *R1407* frameshift in the *SETD2* gene of the PIK3CA pathway, *R462H* in the *NF2* gene, and a *I463V* mutation in *TSC2*. Two patients testing positive for *EGFR* expression were negative for the *EGFRvIII* variant. Herein, we identified several alterations such as those in *BRAF*^*V600E*^ and PD-L1, which may be considered as targets for combination therapy of residual craniopharygiomas.

## Introduction

Craniopharyngioma is a rare, benign but heterogeneous tumor of the pituitary stalk, comprising 1–3% of all brain tumors [1]. It is the most common childhood suprasellar tumor; however, it has a bimodal age distribution and may be observed in adults between age 50 and the late 70s, who are the focus of this manuscript [2]. Two theories have been debated regarding the etiology of craniopharyngioma. The first one proposes that craniopharyngiomas develop from the transformation of oral ectodermal embryologic remnants of the Rathke pouch, whereas the other hypothesis argues that this tumor originates from metaplasia of the primordial adenohypophysis cells [3, 4]. These tumors are typically treated with surgery; however, residual tumor and recurrence can pose a treatment quandary because little is known about the genetic landscape of these tumors beyond two defining mutations: *BRAF V600E* and *CTNNB1* [5, 6].

Papillary craniopharyngioma, primarily seen in adults, is associated with *BRAF V600E* mutation whereas the adamantinomatous type, which is more common in children, is linked to mutations in the ß-catenin gene or a mediator of the Wnt pathway *CTNNB1*; however, both subtypes have been described in adults. Craniopharyngiomas are not histologically malignant, but they often are locally aggressive and can thus cause debilitating visual, endocrine, and neurologic symptoms and a decrease in survival. There are two treatment options available, either attempting an aggressive complete resection, or performing a more conservative resection in preparation for adjuvant radiation therapy. Both options have potential complications, including cerebrovascular injury, neurocognitive decline, and metabolic alterations, including frequent panhypopituitarism [7–10]. Furthermore, the partial resection and radiation therapy combination leaves remnants of the tumor, which can lead to recurrence and repetitive surgical risks, exposes patients to a higher risk of radiation-induced secondary malignancy, and multiple recurrences are associated with malignant transformation [11–13]. Consequently, genetic profiling may provide insight into new therapeutic strategies and a better understanding of the etiology, development and progression of these tumors. As such, we hypothesized that sequencing for cancer hotspot mutations may reveal novel therapeutic targets that could be considered in scenarios where patients have sub totally resected or unresectable craniopharygioma.

## Materials and methods

### Study population

Multiplatform analysis covering the tumor mutational burden (TMB), microsatellite instability (MSI), high-throughput sequencing, *in situ* hybridization, and immunohistochemical study was performed on six craniopharyngioma tumors in adults and identified in the Caris Life Sciences database. The purpose of the database is to provide a genetic profiling record, but annotation of clinical data is limited. As such, the history, treatment, and survivorship outcomes of patients are not included. The histologic diagnosis is based on WHO guidelines (ICD10–2016).

### Genetic analysis

Genomic DNA was extracted from formalin-fixed paraffin-embedded (FFPE) tumor blocks using the QIAamp DNA FFPE DNA Extraction Kit (Qiagen Sciences, Germantown, MD 20874). Genes of interest, cited in Supplementary Table 1, were amplified using the Illumina TruSEQ amplicon cancer hotspot (47 genes; n=1)(Illumina, San Diego, CA) or the Agilent customized pan-cancer panel (592 genes; n=4)(Agilent Technologies, Santa Clara, CA) depending on the availability of both tissue and sequencing panels, with an overlap of the genes in both panels regardless of the size, and sequenced with the Illumina MiSEQ and Illumina NextSEQ platforms, respectively, out of a total of 1.4 megabases of DNA. The analysis focused on the TMB, MSI, and specific gene mutations and their transcriptional effect. TMB was measured by counting all non-synonymous missense mutations found per tumor that had not been previously described as germline alterations, the threshold used for TMB was 17 mutations/megabase based on concordance data with MSI in colorectal cancer. MSI was examined using over 7,000 target microsatellite loci and compared to the reference genome hg19 from the University of California, Santa Cruz (UCSC) Genome Browser database. The threshold to determine MSI by NGS was 46 or more loci with insertions or deletions to generate a sensitivity of > 95% and specificity of > 99%. Variants were detected with a >99% confidence interval based on the frequency of identified mutations and amplicon coverage, with an average coverage of > 500 and an analytic sensitivity of 5%.

### Gene amplification and expression

Both fluorescent and chromogenic *in situ* hybridization were used to detect amplifications in *cMET, Her2* and *cMET* amplifications, respectively, as well as gene fusion of *ALK*. Analysis by immunohistochemistry (IHC) was performed on full FFPE sections to assess the expression of EGFR, Her2/Neu, cMET, PD-L1 and ALK chosen based on the relevance in cancer. Slides were stained using automated techniques, per the manufacturer’s instructions, and were optimized and validated per Clinical Laboratory Improvement Amendments CLIA/CAO and international Organization for Standardization (ISO) requirements. Staining was scored for intensity (0 = no staining; 1+ = weak staining; 2+ = moderate staining; 3+ = strong staining) and staining percentage (0–100%). Results were categorized as positive or negative by defined thresholds specific to each marker based on published clinical literature that associates biomarker status with patient responses to therapeutic agents. For PD-L1, the primary antibody used was SP142 (Spring Biosciences). The staining was regarded as positive if its intensity on the membrane of the tumor cells was >=2+ and the percentage of positively stained cells was >5%. A board-certified pathologist evaluated all IHC results independently. For gene fusion detection, anchored multiplex PCR was performed for targeted RNA sequencing using the ArcherDx fusion assay (Archer FusionPlex Solid Tumor panel). The formalin-fixed paraffin-embedded tumor samples were microdissected to enrich the sample to ≥20% tumor nuclei, and mRNA was isolated, and reverse transcribed into complementary DNA (cDNA). Unidirectional gene-specific primers were used to enrich for target regions, followed by Next-Generation sequencing (Illumina MiSeq platform). Targets included 52 genes, and the full list can be found at http://archerdx.com/fusionplex-assays/solid-tumor.

## Results

### Demographics

The study cohort included six adult patients who were diagnosed with craniopharyngioma. The patients’ ages ranged from 33 to 78 years, with the median age being 54.5 years. Four patients presented with a newly diagnosed craniopharyngioma, and the disease was metastatic in one patient. The mass was in the parasellar in one, in the suprasellar region in two, in the Rathke pouch in one, in the frontal lobe (recurrent) in one, and in an unspecified location in another. Based on histology, three of the tumors were papillary, one adamantinomatous, and two were undefined because of the distorted architecture that does not fall in any of the predefined subtypes implying a possibility of a mixed subtypes or a new distinct phenotype (Table 1).

### Craniopharyngiomas are genomically stable but express PD-L1

**Figure 1:**
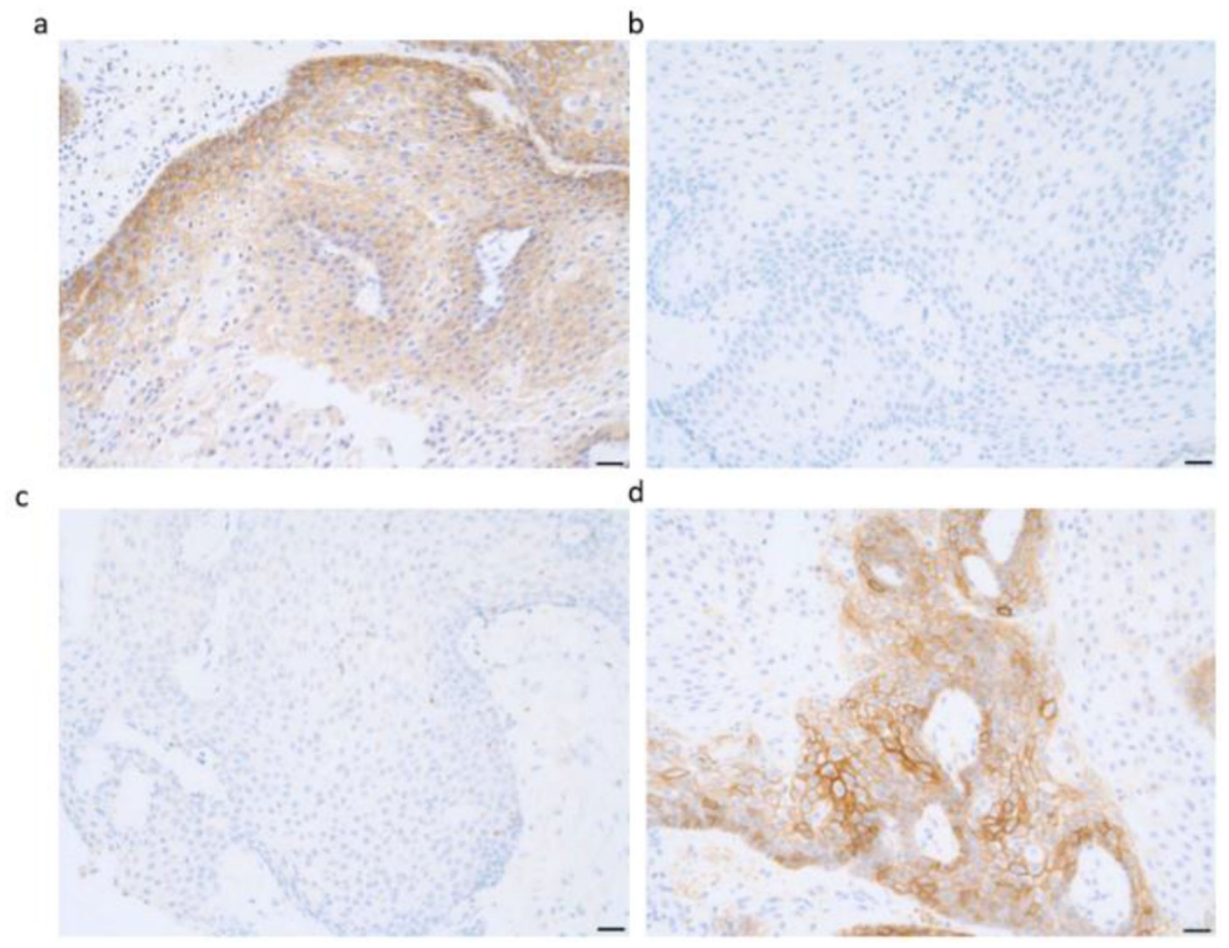
Representative immunohistochemical analysis of (a) the epidermal growth factor receptor (EGFR), (b) Her2, (c) ALK, (d) PDL1 in tumor cells from patient #2. Staining was positive for expression of both the EGFR and PD-L1, but not for Her2 or ALK. (Magnification = 20X in a through d.)

To clarify whether craniopharygiomas expressed biomarkers associated with a potential response to immune checkpoint inhibitors, the tumors were assessed for both MSI and TMB. Of the patients tested (n=4), none showed MSI and all showed a relatively low TMB including the recurrent case (Table 2). No mutations in the DNA repair genes (MLH1, MSH2, MSH6, PMS2) were detected (data not shown). Tumors in four of the five patients profiled were positive for PD-L1 expression, as assayed by IHC at a cut point of 2+ staining intensity of at least 5% cells (Figure 1). All tumors demonstrated some PD-L1 staining.

### Craniopharyngiomas express a variety of mutations with known pathogenic effects

Pathogenic mutations known for craniopharyngiomas are summarized in (Table 2). Four out of six patients had mutations in *BRAF*, specifically the *V600E* missense mutation known to be expressed in the papillary subtype of craniopharyngioma. One patient had a mutation in the WNT pathway, specifically a missense mutation in *CTNNB1* typically associated with adamantinomatous craniopharyngiomas. The same patient with mutation in *CTNNB1* also had a mutation in the *NF2* gene—specifically an *R462H* mutation of unknown significance that may act as a driver. Novel mutations not previously described included an *E318K* missense mutation in the *MITF* gene and an *R1407* frameshift in the *SETD2* gene. One patient had a kinase domain mutation in exon 20 (H1047R) in PIK3CA gene that’s been reported to activate the PI3K/Akt/mTOR pathway.

### Craniopharyngiomas overexpress EGFR

Using fluorescent and chromogenic *in situ* hybridization, we evaluated for amplifications of cMET (n=2) and Her2 (n=3) and no amplifications were seen. ALK FISH was tested on one tumor and no gene fusion was detected. RNA sequencing was done on another two tumors and no gene fusion was detected of the 52 genes interrogated. Gene copy number alteration was also evaluated on 442 of the 592 genes sequenced on the four tumors and no amplification event was seen. Immunohistochemistry on EGFR was done in two tumors and showed overexpression on both (2/2).

## Discussion

To date, there has not been comprehensive sequencing information or extensive immune profiling reported on craniopharyngiomas. Previous craniopharyngioma sequencing studies have only focused on either codon hotspot mutations in *BRAF* and *CTNNB1* or evaluations that were limited to 23- or 46-gene panels [5, 14–17]. Immune profiling is limited to few previous studies [18, 19]. Whole exome sequencing was previously performed on craniopharyngioma, however this does not detect hotspot genes that are directly implicated in cancer [5]. As such, we performed genetic sequencing of 592 genes, gene amplification assessments, and immune profiling analysis on craniopharyngiomas to study the TMB, MSI, and genetic alterations that could be further explored as therapeutic targets. Consistent with prior reports, our study found that the *BRAF*^*V600E*^ mutation was the most common mutation in craniopharygiomas, and we also identified another tumor with a *CTNNB1* mutation with a *G34E* substitution [15]. These two unique mutations have been previously described to occur exclusively in the papillary (*BRAF*^*V600E*^) and adamantinomatous (*CTNNB1*) subtypes, respectively, and were proposed to be driver mutations of their correspondent subtypes; however, their single driver oncogenic potential has been questioned [20, 21]. Despite the relatively low mutational burden seen in craniopharyngiomas, we found several unique mutations, including one in the melanocyte-inducing transcription factor (*MITF*) gene (*E318K*) and another in the *SET* Domain Containing 2 gene (*SETD2*) (*R1407* frameshift). These two mutations have not been previously described in craniopharyngiomas but are associated with other types of tumors. *MITF* (*E318K*) mutation has been associated with neural crest-derived tumors, melanomas, and renal cell carcinomas, whereas the *SETD2* frameshift mutation was previously described in gastrointestinal tumors [22–24]. Histone deacetylase (HDAC)-inhibitor drugs could be considered for treatment in the clinical scenario of upregulated MITF and SETD2 inhibitors are currently being investigated in the treatment of leukemia [25, 26].

The higher the tumor mutational burden is, the more the immune system recognizes the cell as non-self and attacks it. In our study, the levels of TMB and MSI (a condition known as genetic hypermutation) were low, there were no alterations in DNA repair genes, but we did observe expression of the PD-L1 in most samples regardless of the tumor subtype. The utility of a given biomarker such as TMB, MSI, or PD-L1 to correlate with therapeutic response to immune checkpoint inhibitors is lineage dependent and it is unknown if these types of agents would be efficacious for craniopharyngiomas. PD-L1 expression in the stromal fibrovascular core in the papillary subtypes of craniopharygiomas and on the cystic lining in the adamantinomatous subtypes has been previously described [19]. In an attempt to find treatment strategies, Coy et al., specifically looked at overlap between PD-L1 expression and genetic alterations such as *BRAF* papillary and *CTNNB1* mutations. With such substantial overlap between *BRAF* mutations and PD-L1 expression, our combined findings would support consideration of a clinical trial using BRAF/MEK inhibitors in combination with immune checkpoint inhibitors in craniopharyngioma patients with refractory or residual disease and in the neoadjuvant setting prior to radiation therapy. This combination is currently being evaluated for safety and efficacy in melanoma patients ().

Craniopharyngiomas could result from a loss-of-function mutation in a tumor suppressor gene or a gain of function in an oncogene. For loss-of-function mutations, both alleles of a tumor suppressor gene must be lost in order to induce a tumor, unlike the case in oncogenes in which only one allele needs to be mutated. In the current study, we found losses of the neurofibromatosis (*NF*) *type 2* (*R462H*) gene and the tuberous sclerosis type 2 (*I463V*) gene, which have not been previously described. *NF2* alterations have been previously shown to be associated with schwannoma, ependymoma, and meningioma, and tuberous sclerosis with ependymoma [27, 28]. It is unclear what role these two genes may play in the underlying development of craniopharyngioma, including in the rare instance of familial craniopharyngioma, but this is an area for future investigation [29, 30]. Our molecular profiling also showed that the phosphoinositide-3-kinase, catalytic, alpha polypeptide (*PIK3CA*) gene, which is involved in cellular proliferation and inhibition of apoptosis, was mutated in one case. Somatic mutations of PIK3CA are common in a variety of primary tumors such as those of the colon, breast, and stomach [31]. Phosphatidylinositol 3-kinase (PIK3) is known to regulate the tuberous sclerosis (TSC) tumor suppressor gene [32]. Both the *PIK3CA* and the *TSC2* mutations were observed in two patients in our study, suggesting that the roles of *PIK3CA* and *TSC2* merit further investigation as to their contributions to the etiology of craniopharyngioma. mTOR inhibitors could be considered for those patients with TSC2 mutations [33]. The only FDA-approved pan-PIK3 inhibitor is Copanlisib, but it is nonspecific and may have unacceptable toxicity due to off-target effect [34]. Specific PIK3 inhibitors are being employed in clinical trials of advanced stage cancers, and the positive overall response rates and progression-free survival rates being observed for *PIK3CA*-mutant tumors may make this a useful therapeutic strategy for a subset of craniopharygiomas [35, 36].

The epidermal growth factor receptor (EGFR), but not the EGFRvIII variant, is expressed in craniopharygiomas as validated by the IHC, and EGFR upregulation is implicated in cell differentiation, proliferation, apoptosis, and migration of these tumors [37]. Furthermore, EGFR expression has been reported in craniopoharyngioma and EGFR phosphorylation has been shown to enhance adamantinomatous craniopharyngioma cell migration and has been proposed as an escape mechanism for radiation therapy [38, 39]. EGFR inhibitors such gefitinib, erlotinib, and lapatinib are now routine treatments in non-small cell lung cancer and breast cancer and could be considered for off-label use in craniopharygiomas. The response to BRAF inhibitors in papillary craniopharyngioma has shown promise, but the tumor recurs shortly after treatment interruption in most cases [40]. Subsequently, BRAF inhibition combined with the MEK inhibitor trametinib has shown a decrease in proliferation of tumor cells *in vitro* and in preclinical xenograft models and produced a dramatic response in a refractory papillary craniopharyngioma case [41, 42]. This is not entirely surprising because this is an established combination strategy for the treatment of melanoma [43]. However, it is unclear whether the genetic variability that underlies each subtype would uniformly demonstrate clinical benefit, but based on the aforementioned data, a clinical trial of this combination would be justified in the adult craniopharygioma patient population.

We would have liked to profile many more of these cases, as further exploration of several mutations in a larger population is warranted. This is likely to require multicenter efforts and commitment to increase the sample size and increase the power of such extensive sequencing. Another limitation of the current study is that the sequencing was done from FFPE blocks, resulting in low coverage for some of the genes in the panel sequenced, and thereby their exclusion. We also are unable to associate the genetic findings with prognosis nor to conclude whether their roles are as driver mutations. Moreover, we note that many studies currently focus on the adamantinomatous subtype, taking for granted the high frequency of the *BRAF*^*V600E*^ mutation and the availability of BRAF and MEK inhibitors, which have demonstrated marked antitumor activity within the CNS [44]. As such, the current study provides additional justification for the triple combination of BRAF and MEK inhibitors plus immune checkpoint inhibitors.

## Supplementary Material

1

## Figures and Tables

**Table 1:** Craniopharyngioma study demographics

Number of patients (n)	6
Age	
Median, years (range)	54.5(33–78)
Sex	
Male, n (%)	3 (50%)
Female, n (%)	3 (50%)
Primary, n (%)	4 (66.6%)
Recurrent, n (%)	1 (16.7%)
NOS, n (%)	1 (16.7%)
Craniopharyngioma subtype	
Papillary, n (%)	3 (50%)
Adamantinomatous, n (%)	1 (16.7%)
Undefined, n (%)	2 (33.3%)
Location	
Parasellar, n (%)	1 (16.7%)
Suprasellar, n (%)	2 (33.3%)
Rathke pouch, n (%)	1 (16.7%)
Frontal lobe, n (%)	1 (16.7%)
NOS, n (%)	1 (16.7%)

**Table 2:** Patients with craniopharyngioma--mutational profiles

Patient	#1	#2	#3	#4	#5	#6
Microsatellite instability	NS	NS	Stable	Stable	Stable	Stable
Tumor mutational burden (per Mb)	NS	NS	7	8	6	4
**Mutations of known significance**						
BRAF		V600E	V600E	V600E		V600E
CTNNB1					G34E	
MITF			E318K			
PIK3CA	H1047R					
SETD2					R1407fs	
**Oncogenes**						
ALK	NS	NS	WT	WT	WT	WT
BCL2	NS	NS	WT	WT	WT	WT
BRAF	NS	V600E	V600E	V600E	WT	V600E
KIT	NS	NS	WT	WT	WT	WT
MYCN	NS	NS	WT	WT	WT	WT
HER2	NS	NS	WT	WT	WT	WT
JAK2	NS	NS	WT	WT	WT	WT
KRAS	NS	NS	WT	WT	WT	WT
HRAS	NS	Ind	WT	WT	WT	WT
N-ras	NS	NS	WT	WT	WT	WT
**Tumor suppressors**						
APC	NS	NS	WT	WT	WT	WT
BRCA1	NS	NS	WT	WT	WT	WT
BRCA2	NS	NS	WT	WT	WT	WT
CDKN2A	NS	NS	WT	WT	WT	WT
SMAD4	NS	NS	WT	WT	WT	WT
Men1	NS	NS	WT	WT	WT	WT
NF1	NS	NS	WT	WT	WT	WT
NF2	NS	NS	WT	WT	R462H	WT
PTEN	NS	NS	WT	WT	WT	WT
Rb	NS	NS	WT	WT	WT	WT
TP53	NS	NS	WT	WT	WT	WT
TSC1	NS	NS	WT	WT	WT	WT
TSC2	NS	NS	WT	I463V	WT	WT
**Targeted therapy status**						
EGFR	Positive	Positive	NS	NS	NS	NS
PD-L1	NS	Positive	Positive	Negative	Positive	Positive
