# Supplementary material for: Genetic and immune profiling for potential therapeutic targets in adult human craniopharyngioma
Source: Clin Oncol Res. Author manuscript; Available in PMC 2019 Nov 11. (PMC6844364; doi:10.31487/j.COR.2019.03.05)
Supplement: 1 [file NIHMS1057814-supplement-1.pdf]

Supplementary Table 1: List of genes sequenced

BRAF	FANCD2	BCL9	CDKN2C	EML4	FLT1	IKBKE	MDM4	NSD1	PRCC	SH3GL1	TNFAIP3
ABL1	GATA1	BCOR	CDX2	EP300	FLT4	IKZF1	MDS2	NT5C2	PRDM1	SLC34A2	TNFRSF 14
AKT1	MAML2	BCORL1	CHCHD7	EPHA3	FNBP1	IL2	MECOM	NTRK1	PRDM16	SLC45A3	TNFRSF 17
ALK	MRE11	BCR	CHEK1	EPHA5	FOXA1	IL21R	MED12	NTRK2	PRF1	SMAD2	TOP1
APC	MYH11	BIRC3	CHEK2	EPHB1	FOXL2	IL6ST	MEF2B	NTRK3	PRKAR1 A	SMARC A4	TPM3

ATM	PTPRC	BLM	CHIC2	EPS15	FOXO1	IL7R	MAP2K1	NUMA1	PRKDC	SMARCE1	TPM4
KIT	RNF213	BMPR1A	CHN1	ERBB3	FOXO3	INHBA	MAP2K2	NUP214	PRRX1	SNX29	TPR
CDH1	ZNF384	BRCA1	CIC	ERC1	FOXO4	IRF4	MEN1	NUP93	PSIP1	SOCS1	TRAF7
MET	MITF	BRCA2	CIITA	ERCC1	FOXP1	IRS2	MKL1	NUP98	PTCH1	SOX10	TRIM26
CSF1R	ABI1	BRD3	CLP1	ERCC2	FSTL3	ITK	MLF1	NUTM1	RABEP1	SOX2	TRIM27
CTNNB1	ABL2	BRD4	CLTC	ERCC3	FUBP1	JAK1	MLLT1	NUTM2B	RAC1	SPECC1	TRIM33
EGFR	ACKR3	BRIP1	CNBP	ERCC4	FUS	JAZF1	MLLT10	OLIG2	RAD21	SPEN	TRIP11
ERBB2	ACSL3	BTG1	CNOT3	ERCC5	GAS7	JUN	MLLT11	OMD	RAD50	SPOP	TRRAP
ERBB4	ACSL6	BTK	CNTRL	ERG	GATA2	KAT6A	MLLT3	P2RY8	RAD51	SRC	TSC1
FBXW7	AFF1	BUB1B	COL1A1	ESR1	GATA3	KAT6B	AFDN	PAFAH1B2	RAD51B	SRGAP3	TSC2
FGFR1	AFF4	EMSY	COPB1	ETV1	GID4	KCNJ5	MLLT6	PAK3	RAF1	SRSF2	TSHR
FGFR2	AKAP9	C15orf65	COX6C	ETV4	GMPS	KDM5A	MN1	PALB2	RALGDS	SRSF3	TTL
FLT3	AKT2	WDCP	CREB1	ETV5	GNA13	KDM5C	MNX1	PATZ1	RANBP17	SS18	U2AF1
GNA11	AKT3	CACNA1D	CREB3L1	ETV6	GOLGA5	KDM6A	MSH2	PAX3	RAP1GDS1	SS18L1	UBR5
GNAQ	ALDH2	CALR	CREB3L2	EWSR1	GOPC	KDSR	MSH6	PAX5	RARA	SSX1	USP6
GNAS	AMER1	CAMTA1	CREBBP	EXT1	GPC3	KEAP1	MSI2	PAX7	RBM15	STAG2	VEGFA
HNF1A	AR	CANT1	CRKL	EXT2	GPHN	KIAA1549	MSN	PAX8	RECQL4	STAT3	VEGFB
HRAS	ARAF	CARD11	CRLF2	EZH2	ADGRA2	KIF5B	MTCP1	PBRM1	REL	STAT4	VTI1A
IDH1	ARFRP1	CARS	CRTC1	EZR	GRIN2A	KLF4	MTOR	PBX1	RHOH	STAT5B	WAS
JAK2	ARHGAP26	KNL1	CRTC3	FAM46C	GSK3B	KLHL6	MUC1	PCM1	RICTOR	STIL	NSD2
JAK3	ARHGEF12	CASP8	CSF3R	FANCA	H3F3A	KLK2	MUTYH	PCSK7	RMI2	SUFU	NSD3
KDR	ARID1A	CBFA2T3	CTCF	FANCC	H3F3B	KMT2A	MYB	PDCD1	RNF43	SUZ12	WIF1
KRAS	ARNT	CBFB	CTLA4	FANCE	HERPUD1	KMT2C	MYC	PDCD1LG2	ROS1	SYK	WISP3
MLH1	ASPSCR1	CBL	CTNNA1	FANCF	HEY1	KMT2D	MYCL	PDE4DIP	RPL10	TAF15	WRN
MPL	ASXL1	CBLB	CYLD	FANCG	HGF	KTN1	MYCN	PDGFB	RPL22	TAL1	WT1
NOTCH1	ATF1	CBLC	CYP2D6	FANCL	HIP1	LASP1	MYD88	PDGFRB	RPL5	TAL2	WWTR1
NPM1	ATIC	CCDC6	DAXX	FAS	HIST1H3B	LCK	MYH9	PDK1	RPN1	TBL1XR1	XPA
NRAS	ATP1A1	CCNB1IP1	DDB2	FBXO11	HIST1H4I	LCP1	NACA	PER1	RPTOR	TCEA1	XPC
PDGFRA	ATP2B3	CCND1	DDIT3	FCRL4	HLF	LGR5	NBN	PHF6	RUNX1	TCF12	XPO1
PIK3CA	ATR	CCND2	DDR2	FEV	HMGA1	LHFPL6	NCKIPSD	PHOX2B	RUNx1T1	TCF3	YWHAE
PTEN	ATRX	CCND3	DDX10	FGF10	HMGA2	LIFR	NCOA1	PICALM	SBDS	TCF7L2	ZBTB16
PTPN11	AURKA	CCNE1	DDX5	FGF14	HMGN2P46	LMO1	NCOA2	PIK3CG	SDC4	TCL1A	ZMYM2
RB1	AURKB	CD274	DDX6	FGF19	HNRNPA2B1	LMO2	NCOA4	PIK3R1	SDHAF2	TERT	ZNF217
RET	AXL	CD74	DEK	FGF23	HOOK3	LPP	NDRG1	PIK3R2	SDHB	TET1	ZNF331
SMAD4	BAP1	CD79A	DICER1	FGF3	HOXA11	LRIG3	NF1	PIM1	SDHC	TET2	ZNF521
SMARCB1	BARD1	CD79B	DNM2	FGF4	HOXA13	LRP1B	NF2	PLAG1	SDHD	TFE3	ZNF703

SMO	BCL10	CDC73	DNMT3A	FGF6	HOXA9	LYL1	NFE2L2	PML	SEPT5	TFEB	ZRSR2
STK11	BCL11A	CDH11	DOT1L	FGFR1OP	HOXC11	MAF	NFIB	PMS1	SEPT6	TFG	MSI
TP53	BCL11B	CDK12	EBF1	FGFR3	HOXC13	MAFB	NFKB2	PMS2	SEPT9	TFPT	TMB
VHL	BCL2	CDK4	ECT2L	FGFR4	HOXD11	MALT1	NFKBIA	POLE	SET	TFRC	
AFF3	BCL2L11	CDK6	EIF4A2	FH	HOXD13	MAP2K4	NIN	POT1	SETBP1	TGFBR2	
ARID2	BCL2L2	CDK8	ELF4	FHIT	HSP90AA1	MAP3K1	NKX2-1	POU2AF1	SETD2	THRAP3	
AXIN1	BCL3	CDKN1B	ELK4	FIP1L1	HSP90AB1	MAX	NONO	POU5F1	SF3B1	TLX1	
CEBPA	BCL6	CDKN2A	ELL	FLCN	IDH2	MCL1	NOTCH2	PPARG	SFPQ	TLX3	
CLTCL1	BCL7A	CDKN2B	ELN	FLI1	IGF1R	MDM2	NR4A3	PPP2R1A	SH2B3	TMPRSS2	
